# Physiologically distinct neuronal type in primate cortex

**DOI:** 10.18632/oncotarget.4796

**Published:** 2015-07-09

**Authors:** Bo Wang, Yousheng Shu

**Affiliations:** State Key Laboratory of Cognitive Neuroscience and Learning, IDG/McGovern Institute for Brain Research, School of Brain and Cognitive Sciences, the Collaborative Innovation Center for Brain Science, Beijing Normal University, Beijing, China

**Keywords:** Pathology Section

Unlike other physiological systems, the central nervous system (CNS) of primates evolves dramatically from that of other mammal species. Therefore, neuroscientists are concerned that conclusions derived from studies using common model animals, such as rodents, may not apply to higher primates, especially the humans. Even though studies on primates are strongly restricted by limited subject resource and accessibility, they have been much valued across all the extent of neuroscience. One of the promising means to uncover the detailed functional organization of human neuronal microcircuit is to take advantage of the discarded brain tissue during neurosurgery and directly perform electrophysiological recording from human neurons. Differences in neuronal cell type or synaptic connectivity pattern between CNS circuits of higher and lower mammals, qualitatively and/or quantitatively, would be no doubt one of the most exciting findings in the field of neuroscience.

**Figure 1:**
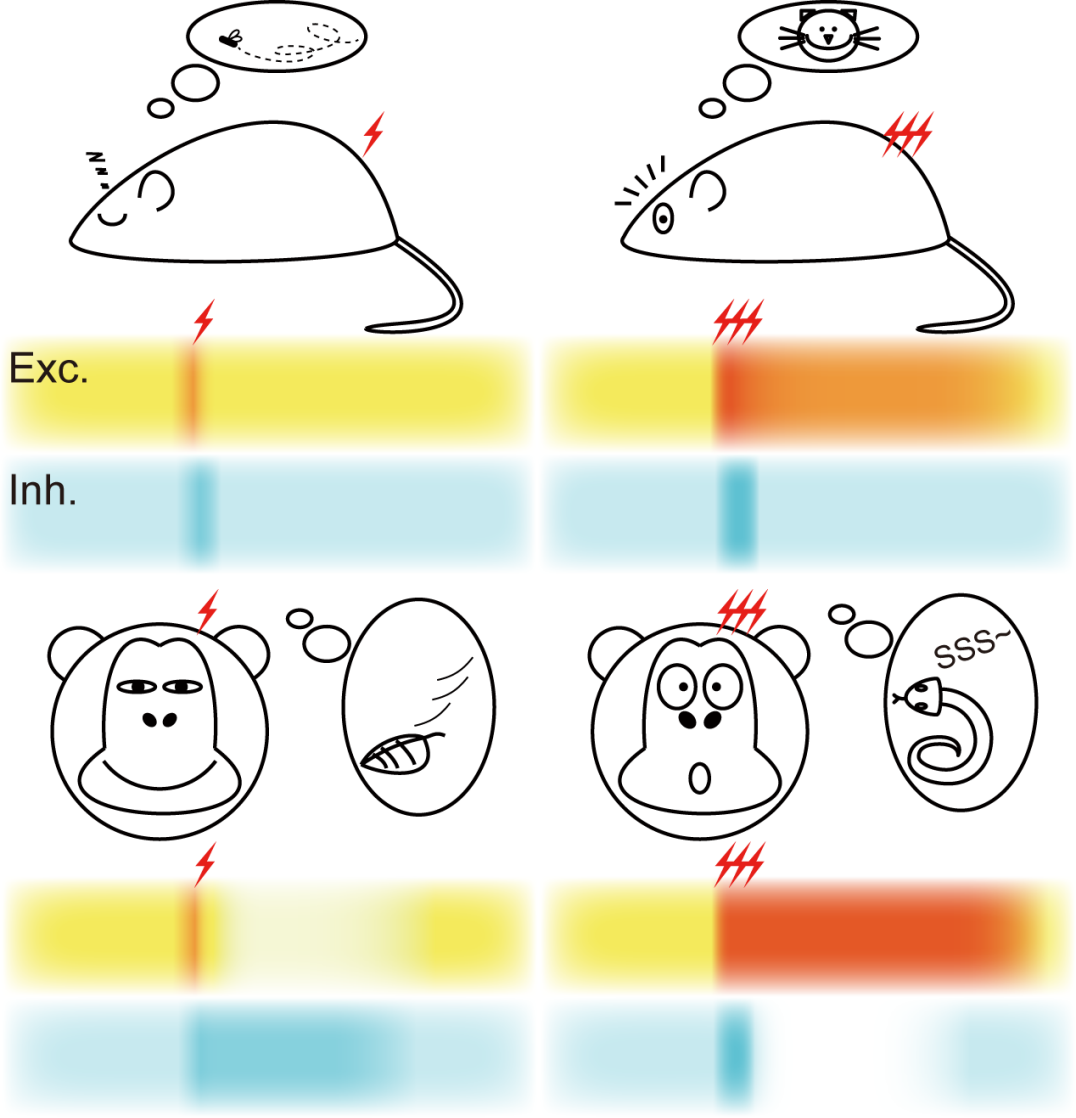
Schematic drawing showing the hypothesized behavior relevance of PANs in primates In rodents (top), neocortical excitatory neurons (Exc.) generate persistent activity when they receive strong inputs. With the contribution of PANs in primates (bottom), weak inputs cause long-lasting inhibition (Inh.) in excitatory neurons, while strong inputs inhibit the activity of PANs and facilitate the persistent activity in excitatory neurons, resulting in an increase in the discriminative ability of primate neurons.

In our recent study, we reported a subgroup of human and monkey cortical inhibitory interneurons possessing distinct intrinsic physiological features, but similar features were not found in rodent neocortical neurons [1]. Those neurons could fire a train of persistent action potentials (APs) after each brief single-AP stimulus, thus we named them persistent-activity neurons (PANs). Stimulation-triggered persistent AP firing is quite widely found in central neurons [2, 3]. However, PANs we found in human and monkey neocortex are distinct in two aspects. First, unlike other forms of persistent activity that require prolonged burst of APs, the persistent firing in PANs is triggered by brief but stopped by strong stimuli. Second, the mechanism for the generation of persistent firing in PANs is attributed to the activation of a persistent Na^+^ current without the involvement of metabotropic neurotransmitter receptors. We speculate that PANs may contribute to distinct physiological functions in primate brain. Since PANs are inhibitory interneurons, they are most likely responsible for controlling the responsiveness of principal cells by providing inhibitory tone in local circuits. Hypothetically, cortical neurons discriminate between strong and weak stimuli, putatively behavior relevant and irrelevant, by not only the firing frequency during the stimulation but also the following persistent activity. In rodent brain, strong stimuli can trigger long-lasting depolarizing plateau or persistent activity in principal cells, which may elevate their responsiveness to incoming synaptic inputs arrived right after the stimulation. In contrast, with the help of persistent activity in PANs, the responsiveness of principal cells in primate neocortex could be decreased by long-lasting inhibitory tone after a weak stimulus; the cessation of PAN's persistent activity induced by a relatively strong stimulus, however, would increase the responsiveness of principal cells. Thus, PANs may help promote the discriminative ability of primate cortex (Figure 1).

The experimental results provide evidence that neuronal types with distinct physiological properties exist in our human brain, and it is possible that they may contribute to unique human brain functions. However, there remains a huge gap between these unique neuronal properties and the higher-order cognitive functions. For example, it is still unclear whether PANs are providing inhibition or disinhibition to neighboring principal cells. Cortical disinhibitory tone has been demonstrated recently playing important and distinct roles in cortical functions. Future studies may focus on unveiling the local synaptic connectivity of PANs and their contribution to the operation of local circuits. It is also important to investigate the function of PANs *in vivo* in non-human primates. But first we need to identify some molecular markers that can help in targeting group of PANs in animal models. Single-cell RT-PCR in our experiment could only reveal whether PANs express known markers of cortical neurons, future studies using single-cell transcriptome together with patch-clamp recording [4] may help in identifying new specific markers for PANs.

To search for new cell types in the human neocortex, previous studies focusing on distinct morphological features turn out to be unfruitful, though important findings including the observation of spindle neurons (also known as Von Economo neurons) have been reported [5, 6]. Our study, however, opens a new avenue in searching for unique cell types in human brain by paying attention to their distinct physiological properties. The human brain tissues removed during neurosurgery could be very promising resources for studying the substrates of higher-order brain functions. Though unique features of human neurons are the most eye-catching, findings proving consistence between human and lower animals are, to some extent, no less valuable. For example, a recent study from our lab demonstrated that asynchronous GABA release in human neocortex was enhanced in epileptic conditions, and this pathological change was consistent with that occurred in animal disease model [7], greatly promoting the clinical significance of the findings. Together, our studies and those of others using human brain tissues provide direct quantitative morphological and physiological measurements of human neurons and their circuits. We believe the results are very important for comprehensive understanding and reconstruction of human intelligence.
